# A Perovskite Photovoltaic Mini-Module-CsPbBr_3_ Photoelectrochemical Cell Tandem Device for Solar-Driven Degradation of Organic Compounds

**DOI:** 10.1021/acsenergylett.3c01361

**Published:** 2023-10-02

**Authors:** Seul-Yi Lee, Patricio Serafini, Sofia Masi, Andrés F. Gualdrón-Reyes, Camilo A. Mesa, Jhonatan Rodríguez-Pereira, Sixto Giménez, Hyo Joong Lee, Iván Mora-Seró

**Affiliations:** † Department of Chemistry and Research Institute of Physics & Chemistry, Jeonbuk National University, Jeonju 561-756, South Korea; ‡ Institute of Advanced Materials, Universitat Jaume I, 12071 Castelló de la Plana, Spain; § Facultad de Ciencias, Instituto de Ciencias Químicas, Isla Teja, Universidad Austral de Chile, Valdivia 5090000, Chile; ∥ Center of Materials and Nanotechnologies, Faculty of Chemical Technology, University of Pardubice, Nam. Cs. Legii 565, 53002 Pardubice, Czech Republic; ⊥ Central European Institute of Technology, Brno University of Technology, Purkynova 123, 612 00 Brno, Czech Republic

## Abstract

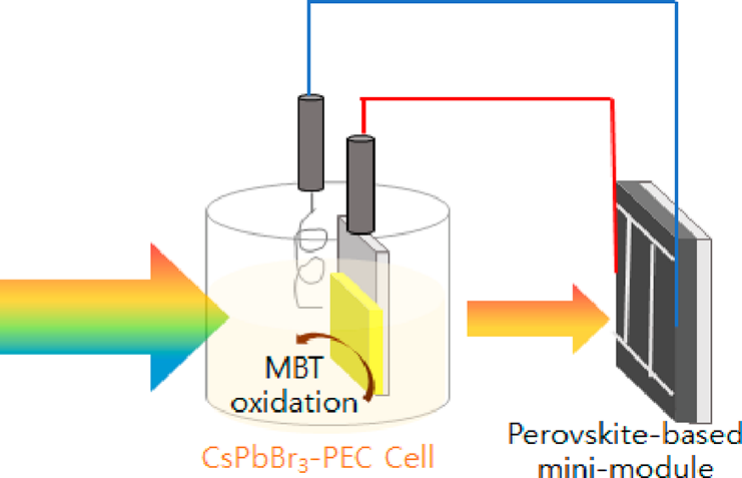

Recently, halide perovskites have been widely explored for high-efficiency photocatalysis or photoelectrochemical (PEC) cells. Here, in order to make an efficient photoanode electrode for the degradation of pollutants, concretely 2-mercaptobenzothiazole (MBT), nanoscale cesium lead bromide (CsPbBr_3_) perovskite was directly formed on the surface of mesoporous titanium dioxide (meso-TiO_2_) film using a two-step spin-coating process. This photoelectrode recorded a photocurrent of up to 3.02 ± 0.03 mA/cm^2^ under standard AM 1.5G (100 mW/cm^2^) illumination through an optimization process such as introducing a thin aluminum oxide (Al_2_O_3_) coating layer. Furthermore, to supply high voltage for efficient oxidation of MBT without an external bias, we developed a new photovoltaic/PEC tandem system using a methylammonium lead iodide (MAPbI_3_) based mini-module consisting of three solar cells interconnected in series and confirmed its successful operation. This approach looks very promising due to its applicability to various PEC reactions.

Energy production based on fossil fuels has caused many environmental issues, such as global warming and the release of pollutants. This has prompted many scientists to become interested in developing clean and sustainable new energy sources. Among them, solar energy is recognized as having the potential to replace conventional fossil fuels, because it is abundant enough to meet the global energy demand.^1^ Recently, very promising results on halide perovskites in various solar energy conversion technologies have been reported exponentially. In particular, the use of lead halide perovskites in solar cells has resulted in very high photovoltaic conversion efficiencies of over 26%^2^ due to their high light-harvesting efficiency, excellent charge transport, defect tolerance, and easily tunable band gap. These outstanding optoelectronic properties have extended lead halide perovskite applications to photoelectrochemical (PEC) cells and photocatalysis.^3−9^

PEC systems, compared to photocatalytic systems, have the advantages of facilitating charge separation and collection as well as catalyst recycling by using photoelectrodes and an external bias.^7^ In the PEC cells, the photogenerated electrons and holes at the photoelectrode are separated and move along their respective paths, leading to various oxidation or reduction reactions, such as water splitting,^5,6,10−13^ degradation of pollutants,^14^ functionalization of C–H bonds,^15^ and CO_2_ reduction,^16^ at the electrode–electrolyte interface. To design an effective PEC system, it is crucial to first find a light absorber with the following conditions: (1) it should be able to absorb light in the visible range, with a band gap energy of 1.5–2.5 eV, (2) the conduction band (CB) minimum and valence band (VB) maximum levels should provide the thermodynamic driving force to allow the desired reaction, and (3) it should possess sufficient stability in the electrolyte solution to ensure its long-term performance and durability. Keeping these conditions in mind, cesium lead bromide, CsPbBr_3_, halide perovskite has recently emerged as a promising candidate for PEC reactions due to its excellent photoelectrical properties and promising robustness.^17^ Some of us already demonstrated that colloidal CsPbBr_3_ nanocrystals (NCs) synthesized by a hot-injection method exhibit a favorable energy band gap for hole injection to 2-mercaptobenzothiazole (MBT) pollutant.^18^ MBT is an organosulfur compound and is used in various industrial areas such as sulfur vulcanization of rubber, fungicides, and herbicides.^18−20^ However, it is considered a potential human carcinogen and is known to be difficult to biodegrade.^18,21^ To investigate the PEC behavior of CsPbBr_3_ NCs in the MBT oxidation reaction, a CsPbBr_3_ NC film based photoanode was made by spin-coating of a CsPbBr_3_ NC solution onto a very thin and compact titanium dioxide (TiO_2_) film, and it showed a photocurrent of about 120 μA/cm^2^ just as a proof-of-concept demonstration,^18^ where the photocurrent was partially limited by the flat configuration of the substrate. In this study, in contrast to conventional perovskite bulk or NC films, the nanoscale CsPbBr_3_-sensitized photoelectrode was prepared by a two-step direct spin-coating of NC precursors onto a mesoporous-TiO_2_ (meso-TiO_2_) film, looking for an increase of effective surface by the use of a mesoporous electrode and an effective decoration of it by the direct growth of a halide perovskite on the TiO_2_ surface. This is a very simple strategy, used in sensitized systems, to form effective nanoscale CsPbBr_3_ photosensitizers directly on the surface of meso-TiO_2_ film, and it allowed us to boost the photocurrent by more than 1 order of magnitude, achieving a high photocurrent of 2.34 ± 0.08 mA/cm^2^ for the MBT oxidation by decoupling the actions of light absorption and charge transport. In the current structure of the CsPbBr_3_-sensitized electrode, the mesoporous metal oxide has the effect of increasing the surface area and improving charge separations and transport through electron injection into meso-TiO_2_, to enhance photocurrents. This approach has already been proven in molecular dye or metal chalcogenide quantum dot sensitized solar cells.^22−24^ However, this perovskite sensitized type has not been applied to PEC electrodes so far but has been used in a few successful examples in solar cells recently.^25−29^ Thus, it looks very promising and timely to test the in situ deposited nanoscale CsPbBr_3_ as a photosensitizer for the target reaction in the PEC cells. As a further step to minimize the defect sites and improve the stability of the as-prepared CsPbBr_3_, a very thin layer of aluminum oxide (Al_2_O_3_) has been deposited over the surface of the meso-TiO_2_/nano-CsPbBr_3_ photoanode by an atomic layer deposition (ALD) technique and its passivation effect in the PEC system for oxidation of MBT was investigated and compared with those in previous studies.^7,14,30,31^

In addition, for an external bias free unassisted PEC reaction of MBT oxidation, a novel photovoltaic (PV)/PEC tandem device was devised by combining methylammonium lead iodide, MAPbI_3_, a perovskite-based mini-module, and the meso-TiO_2_/nano-CsPbBr_3_ PEC system. In the well-known PV/PEC tandem system for water splitting, the photoinduced carriers (electrons or holes) are driven to the counter electrode through the PV cell to drive one of the half-reactions of water splitting, while the other carrier contributes to the complementary half-reaction.^32^ In this tandem system, the PV device provides the photovoltage that drives the water-splitting reaction in the PEC system.^32−34^ In the case of our tandem device for the photodegradation of MBT, electrons and holes are photogenerated at the meso-TiO_2_/nano-CsPbBr_3_ PEC electrode, and then the electrons are driven to the counter electrode through the mini-module, while the holes are responsible for the MBT oxidation. The mini-module fabricated by interconnecting three solar cells in series was used to supply enough voltage (>1.5 V) to lead to the desired MBT oxidation, and its role was confirmed by electrochemical measurements.

The nanoscale CsPbBr_3_-sensitized photoanode was fabricated as described in Figure 1a. 0.3 M lead(II) bromide (PbBr_2_) with the same amount of 4-*tert*-butylpyridine (tBP) in *N*,*N*-dimethylformamide (DMF) and 0.03 M cesium bromide (CsBr) in methanol were used as precursor solutions, and they were sequentially spin-coated onto a meso-TiO_2_ film with a thickness of approximately 1.6 μm. A relatively low concentration of PbBr_2_ (0.3 M) was used compared to the high concentration (>1.0 M) required for CsPbBr_3_ bulk films^29^ (see the Supporting Information for further experimental details). The utilization of such a low concentration of precursors over a meso-TiO_2_ film could enable the direct formation of a nanoscale CsPbBr_3_ perovskite on the surface of the TiO_2_ particulate film, a method that has been successfully proved in our recent works on nanoscale MAPbI_*x*_Br_3–*x*_- or CsPbI_*x*_Br_3–*x*_-sensitized solar cells.^28,35^ In this study, tBP was added to the PbBr_2_ solution to enhance the crystalline quality of the CsPbBr_3_ nanocrystals for optimal performances. The role of tBP is well-known on perovskites made by a 2-step deposition process, and it promotes a reaction with the second precursor by weakening the crystallinity of the first-deposited lead halide.^36,37^ The meso-TiO_2_/nano-CsPbBr_3_ PEC electrode was completed by heating at 280 °C for crystallization of CsPbBr_3_, and then an expected yellow electrode was obtained, as shown in Figure 1a.

**Figure 1:**
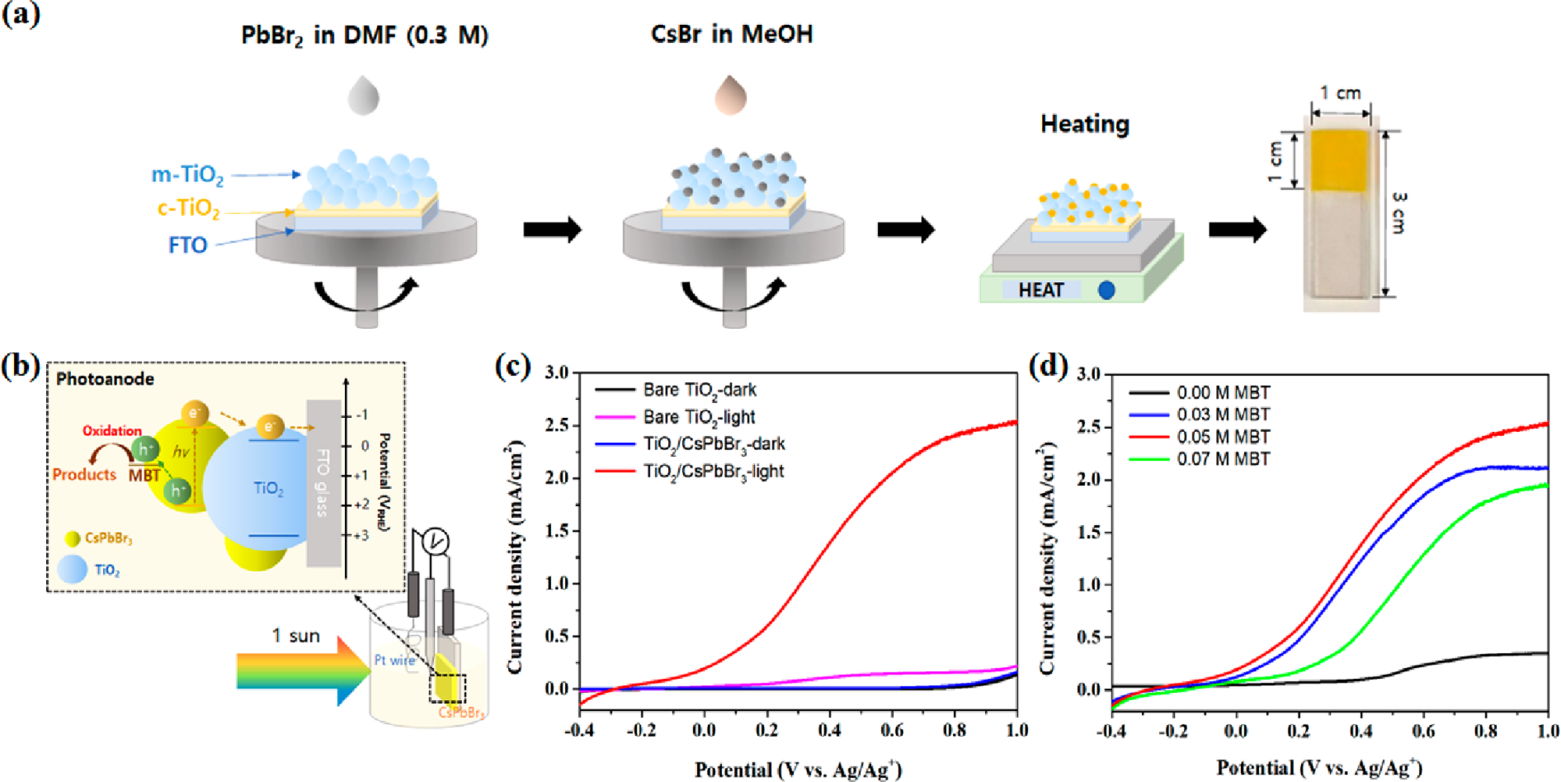
(a) Scheme of the two-step deposition process showing the direct formation of CsPbBr_3_ nanocrystals on the meso-TiO_2_ film with an active area of 1.0 cm^2^ and (b) its application to the PEC cell for MBT oxidation. (c) Linear sweep voltammograms (LSVs) of bare meso-TiO_2_ and meso-TiO_2_/nano-CsPbBr_3_ photoanodes in 0.1 M tetrabutylammonium hexafluorophosphate (Bu_4_NPF_6_) in dichloromethane (DCM) with 0.05 M MBT. (d) LSVs of the meso-TiO_2_/nano-CsPbBr_3_ photoanode depending on the concentration of MBT in the electrolyte. All LSVs were obtained in a three-electrode configuration under AM 1.5G (100 mW/cm^2^) illumination.

To investigate the PEC performances for MBT oxidation, the photocurrents induced by the degradation of MBT were checked under various conditions using a configuration of three electrodes (Figure 1b); a meso-TiO_2_/nano-CsPbBr_3_ photoanode, a nonaqueous Ag/Ag^+^ electrode, and a platinum (Pt) wire were used as the working, reference, and counter electrodes, respectively. MBT is known to be oxidized between 0.47 and 1 V vs NHE depending on the experimental conditions.^38^ As shown in Figure 1b, CsPbBr_3_ perovskites possess a valence band position suitable for injecting holes into MBT, allowing the easy degradation of MBT under illumination conditions. In our previous work, the band positions of CsPbBr_3_ nanocrystals were determined by cyclic voltammetry and the total degradation of MBT was confirmed from the clear disappearance of the initial *m*/*z* (167.9937) characteristic peak provided by electrospray mass spectroscopy (ESI-MS) analysis.^18^ Indeed, upon comparison of the photocurrents of bare meso-TiO_2_ and meso-TiO_2_/nano-CsPbBr_3_ electrodes in an electrolyte of 0.1 M tetrabutylammonium hexafluorophosphate (Bu_4_NPF_6_) in dichloromethane (DCM) containing 0.05 M MBT (Figure 1c), almost no photocurrent was observed in either electrode in the dark. However, under AM 1.5G (100 mW/cm^2^) illumination, the bare meso-TiO_2_ electrode exhibited a very low photocurrent of 0.25 ± 0.06 mA/cm^2^, while the meso-TiO_2_/nano-CsPbBr_3_ photoelectrode showed a significantly higher photocurrent of 2.34 ± 0.08 mA/cm^2^ at 0.8 V (V vs Ag/Ag^+^). All photocurrents were compared at 0.8 V (V vs Ag/Ag^+^), where the highest photocurrent value was observed while the influence of dark current was minimized. This result demonstrates that the photocurrents are primarily coming from visible-light-absorbing CsPbBr_3_, not the meso-TiO_2_ film, though the latter is UV-light absorbing and could contribute to MBT oxidation to some degree. Figure 1d represents the change in photocurrent with the concentration of MBT using a meso-TiO_2_/nano-CsPbBr_3_ electrode. The photocurrent also increased when the concentration of MBT increased from 0.00 to 0.05 M but decreased at 0.07 M. This indicates that an increase of reactant concentration in the electrolyte leads to enhanced kinetics for photocurrent generation. However, beyond a certain threshold, an excessive reactant concentration actually blocks the mesoporous structure and slows down the reaction kinetics. A plausible hypothesis, considering the results of measurements with and without stirring shown below (see Figure 3d for details), is that the presence of a diffusion-controlled approach to the active site at the interface inside the mesostructure dominates until 0.05 M, but after that point, oxidized products come out too slowly from the mesostructure and accumulate for interference, which leads to a decrease of oxidation current. The averaged photocurrent values and their values corresponding to each MBT concentration were obtained using three different photoanodes to check the reproducibility, and they are summarized in Table S1. PEC electrodes made of bulk films often exhibit different photocurrent values depending on the direction of the incident light.^39−41^ Typically, when light enters through the glass side (back side illumination), even though the glass absorbs a certain portion of light, electron transfer to the FTO glass is facilitated, resulting in higher photocurrent values compared to the situation when light enters through the light absorber (front side illumination), indicating charge transport limitations.^41^ However, in the case of this meso-TiO_2_/nano-CsPbBr_3_ PEC electrode, it shows nearly similar photocurrent values regardless of the direction of the incident light (see Figure S1 and Table S2), pointing out the absence of electron transport limitation through the meso-TiO_2_ film. This fact allows one to take full advantage of the increased contact area with the electrolyte, which facilitates efficient hole transfer to MBT from in situ deposited nanoscale CsPbBr_3_ photosensitizers without any long molecular ligands. This, in turn, contributes to the generation of overall high photocurrents exceeding 2.0 mA/cm^2^.

To confirm the influence of the thickness of the meso-TiO_2_ layer on the PEC performance, meso-TiO_2_ films with different thicknesses were produced by diluting a commercially available TiO_2_ paste in different volumes of ethanol. Generally, it is expected that a thicker meso-TiO_2_ film allows the immobilization of a higher fraction of light sensitizers, leading to higher photocurrents. However, when the electrode thickness exceeds the electron diffusion length, the photocurrent decreases.^42^ In this study, it was confirmed that a meso-TiO_2_ thickness of about 1.6 μm was optimal (see Figure S2). Therefore, a meso-TiO_2_ film with this thickness was used in all of the measurements shown hereafter. As shown in Figure 2, the morphology of the optimized meso-TiO_2_/nano-CsPbBr_3_ film looks like that of the bare meso-TiO_2_ film in the scanning electron microscopy (SEM) images of the surface and cross-section. However, transmission electron microscopy (TEM) measurements reveal the presence of a few nanometer-sized CsPbBr_3_ photosensitizers on the surface of the larger TiO_2_ particles. This observation is consistent with the results of our previous work conducted using a similar fabrication method.^35^

**Figure 2:**
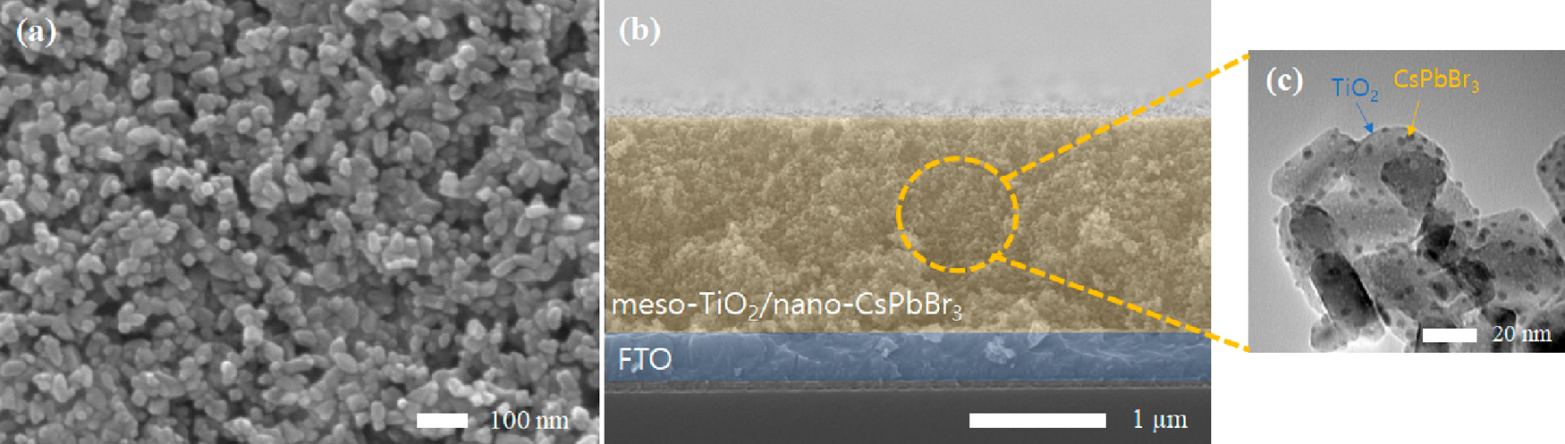
(a) Top-view and (b) cross-section SEM images and (c) TEM image of the meso-TiO_2_/nano-CsPbBr_3_ electrode.

In order to further increase the performance of the system, an ultrathin Al_2_O_3_ layer has been utilized in many solar energy conversion devices to protect the light-absorber material and minimize charge recombination at the interfaces. It has primarily been applied using the well-known atomic layer deposition (ALD) method.^7,30,31^ This method allows the precise control of the thickness of the Al_2_O_3_ layer deposited on all of the effective surface of the target material. It has been widely used not only in bulk film type electrodes^7,31^ but also in mesoporous electrode structures.^30^ To check the passivation effect of Al_2_O_3_ on the PEC system for MBT oxidation, a very thin Al_2_O_3_ layer was applied to the surface of the meso-TiO_2_/nano-CsPbBr_3_ film by the ALD process as an increment of ∼0.9 Å thickness per cycle, and the photocurrent was measured as a function of the Al_2_O_3_ layer thickness (Figure 3a). The photocurrent increased with the thickness of the Al_2_O_3_ layer, and a remarkably high photocurrent of 3.02 ± 0.03 mA/cm^2^ was recorded for samples prepared with 3 cycles (see Table S3). However, higher thicknesses (4 ALD cycles) led to a decrease of the photocurrent (2.71 ± 0.06 mA/cm^2^) due to the higher transfer resistance induced by the Al_2_O_3_ coating layer and the catalytic deactivation of the passivated surface, eventually inhibiting hole transfer to MBT. Figure 3b,c shows the absorbance spectra and X-ray diffraction (XRD) patterns, respectively, before and after 3 cycles of Al_2_O_3_ ALD. Both results match the previously reported optical properties and XRD main peaks of CsPbBr_3_,^31,43,44^ demonstrating that the deposition process shown in Figure 1a is suitable for the formation of CsPbBr_3_. In addition, the lack of any significant changes in the absorbance spectra and XRD peaks corresponding to CsPbBr_3_ after 3 cycles of Al_2_O_3_ ALD confirms that the deposition process of Al_2_O_3_ did not affect the crystalline and optical properties of CsPbBr_3_. However, it is difficult to identify peaks related to Al_2_O_3_ in the XRD pattern (Figure 3c) due to its ultrathin character (about ∼0.27 nm) and/or its amorphous character. To demonstrate the presence of the Al_2_O_3_ layer, X-ray photoelectron spectroscopy (XPS) analysis was performed, and the results clearly showed peaks related to Al_2_O_3_ in the Al 2s and the O 1s spectra (see Figure S3). To further investigate the effect of the thin Al_2_O_3_ layer on the PEC performance, chronoamperometry (CA) measurements were conducted using CsPbBr_3_/Al_2_O_3_(0) (without the Al_2_O_3_ layer) and CsPbBr_3_/Al_2_O_3_(3) (3 cycles with Al_2_O_3_ ALD) photoanode electrodes in the electrolyte with 0.05 M MBT. Both electrodes generated reversible photocurrent upon the on/off illumination cycles, with CsPbBr_3_/Al_2_O_3_(3) initially showing a higher photocurrent compared to CsPbBr_3_/Al_2_O_3_(0). However, both exhibited similar photocurrents over time (Figure S4). This behavior became more evident when the measurement time was extended to 10 min (Figure 3d). In the absence of stirring, the photocurrent of CsPbBr_3_/Al_2_O_3_(3) started at a higher value than that of CsPbBr_3_/Al_2_O_3_(0) but decreased more rapidly. However, with stirring, the photocurrent of CsPbBr_3_/Al_2_O_3_(3) showed higher stability compared to that of CsPbBr_3_/Al_2_O_3_(0). To understand this phenomenon, the evolution of the photocurrent with the MBT concentration was measured without stirring. Figure S5a shows that the decrease in photocurrent is slower at an MBT concentration of 0.08 M compared to 0.05 M. This is because the consumption rate is faster than the supply rate of the reactant, MBT, at the PEC electrode surface. Therefore, CsPbBr_3_/Al_2_O_3_(3), which shows a higher photocurrent, can decompose MBT more rapidly than CsPbBr_3_/Al_2_O_3_(0), leading to a faster decrease in photocurrent. However, when we decrease the mass transport limitation, enhancing the supply of MBT to the electrode surface by stirring, the passivation effect of Al_2_O_3_ enables more stable MBT oxidation. This is further supported by the lower intensity of the absorption peak at around 320 nm corresponding to MBT in the absorbance measurement of the electrolyte after a stability test of 30 min using CsPbBr_3_/Al_2_O_3_(3) with stirring (Figure S5b). Also, from the decrease of the MBT absorption peak after PEC oxidation, the removal efficiency of MBT could be estimated to be about 21% from the initial concentration of 0.05 M by using Beer’s law. This result looks promising because we have used a relatively high concentration (0.05 M) of MBT when compared to a common value of a few or fewer micromoles employed in most degradation experiments by photocatalysts. Moreover, a larger cell volume was used here rather than the typical small volume of a cuvette, and most parameters were not optimized because we focused on the degree of maintaining PEC photocurrents in a relatively high concentration of pollutants by a newly designed electrode with perovskite sensitizers, not on the pollutant removal efficiency. Without stirring, the calculated removal efficiency was about 14% and was not as effective as in the case of stirring. But, when the measurement time was extended to 30 min, the photocurrent gradually decreased even with stirring (Figure S5c). Furthermore, when the electrolyte contained 0.08 M MBT, the effect of stirring was not observed since the concentration of MBT on the electrode surface was already sufficient, avoiding a mass diffusion limitation (Figure S5d). To check what happened to two-step spin-coated nanoscale CsPbBr_3_ after the PEC operations, XRD patterns and photos of FTO/TiO_2_/CsPbBr_3_/Al_2_O_3_ photoanode after a 30 min stability test were obtained, as shown in Figure S6. From these results, we could estimate that CsPbBr_3_ nanoparticles deposited on the electrode were partially detached and some remaining parts were changed to CsPb_2_Br_5_ during the stability test for 30 min. Thus, it seems to be reasonable to conclude that the very thin layer of Al_2_O_3_ applied could not fully protect CsPbBr_3_, but some uncovered parts degraded gradually during MBT oxidation in a less polar solvent, DCM. At the current stage, though this durability in PEC cell looks encouraging for further enhancement, it will be necessary to do more careful checks to extend the working time to a few hours in the next step to higher stability.

**Figure 3:**
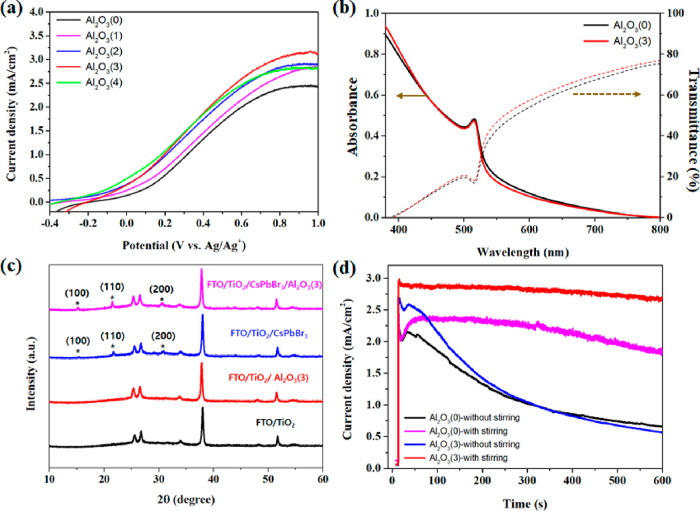
(a) LSVs of meso-TiO_2_/nano-CsPbBr_3_ electrodes prepared with different cycles of Al_2_O_3_ ALD. (b) Absorbance spectra and transmittance of CsPbBr_3_/Al_2_O_3_(0) and CsPbBr_3_/Al_2_O_3_(3) films. (c) XRD patterns of FTO/TiO_2_ and FTO/TiO_2_/CsPbBr_3_ without and with the 3 cycles of Al_2_O_3_ ALD (the main peaks of CsPbBr_3_ in XRD patterns are marked with asterisks). (d) Chronoamperometry (CA) of CsPbBr_3_/Al_2_O_3_(0) and CsPbBr_3_/Al_2_O_3_(3) photoanodes with and without stirring at 0.8 V (V vs Ag/Ag^+^). The LSV and CA measurements were performed in the electrolyte with 0.05 M MBT by using a three-electrode configuration under standard AM 1.5G illumination.

To drive the unassisted photodegradation of MBT in a PEC system without an external bias, a photovoltaic mini-module was used as the voltage supply source. The mini-module was fabricated by interconnecting three solar cells in series based on MAPbI_3_ perovskite, following the architecture shown in Figure S7. The series interconnection of the solar cells does not significantly affect the overall current, but compared to single solar cells, higher voltages are possible, since the total voltage is determined by the sum of each active layer.^45−49^ The characteristics of this mini-module are compatible to those of our PEC system, where a high voltage of at least 1.5 V is required and the photocurrent of the mini-module does not limit the MBT degradation. The combination of the PEC system and the mini-module can have two possible different configurations. The first configuration is a serial tandem one where a narrow-band-gap MAPbI_3_ mini-module is placed behind the wider-band-gap CsPbBr_3_-PEC electrode, allowing light to pass through CsPbBr_3_ and enter MAPbI_3_ (Figure 4a), both systems consequently sharing the 1 sun incident light. Consequently, most of the short-wavelength radiation is absorbed by the electrode while the long-wavelength radiation is mostly absorbed by the mini-module. In the second configuration, the PEC electrode and the mini-module are placed parallel to each other for incident light harvesting (Figure 4d). Consequently, in this configuration the electrode and mini-module both are illuminated with the full 1 sun spectra. The former can be called the tandem serial illumination mode (mode S) and is generally used in various PV/PEC tandem devices^32−34^ due to its efficient light utilization and minimal space constraints. The latter looks similar to the parallel illumination mode (mode P) of a PEC tandem cell.^32,50^ When the mini-module and the CsPbBr_3_-PEC cell are arranged in mode P, each device is able to utilize its maximum efficiency, since both devices are exposed to the same light irradiation. However, a higher effective area for light incidence is required. To verify the operation of the tandem device in mode S, a mask with the same active area as the CsPbBr_3_-PEC cell was used on the mini-module, ensuring that the light passes through both devices with the same area. To achieve high voltage in the mini-module, it is necessary to illuminate all three active layers connected in series. Therefore, the mask was placed so that all three active layers fit within the mask area coinciding with the area of the CsPbBr_3_-sensitized electrode. To predict the operating point of the mini-module/CsPbBr_3_ PEC cell tandem device arranged in mode S, the current density–potential (*J*–*V*) curve of the CsPbBr_3_ PEC cell was obtained by using a two-electrode configuration (Figure 4b). For the *J*–*V* curve of the mini-module, the CsPbBr_3_ film was placed in front of the mini-module like a filter to simulate mode S (the blue line in Figure 4b). Figure 4c shows the current density (∼0.8 mA/cm^2^) obtained from the tandem device in mode S, showing a good match with the operating point predicted from Figure 4b. Samples with and without alumina coating, with a higher performance for the former, were analyzed (Figure 4b,c). To check the performance of the tandem device in mode P, a different MAPbI_3_ perovskite mini-module was used without a mask, and a current density of over 1.0 mA/cm^2^ was obtained (Figure 4e,f). Here, we focused more on the optimization process of the CsPbBr_3_ photoanode electrode for more efficient MBT oxidation. Additional efforts to optimize mini-modules are beyond the scope of this work, as even mini-modules with a low photocurrent are sufficient to avoid current limitation and produce the proof of concept presented here in terms of reaching the necessary photovoltage. Therefore, it is expected that photocurrent enhancement can be achieved through a further optimization process of the integrated PV/PEC architecture.

**Figure 4:**
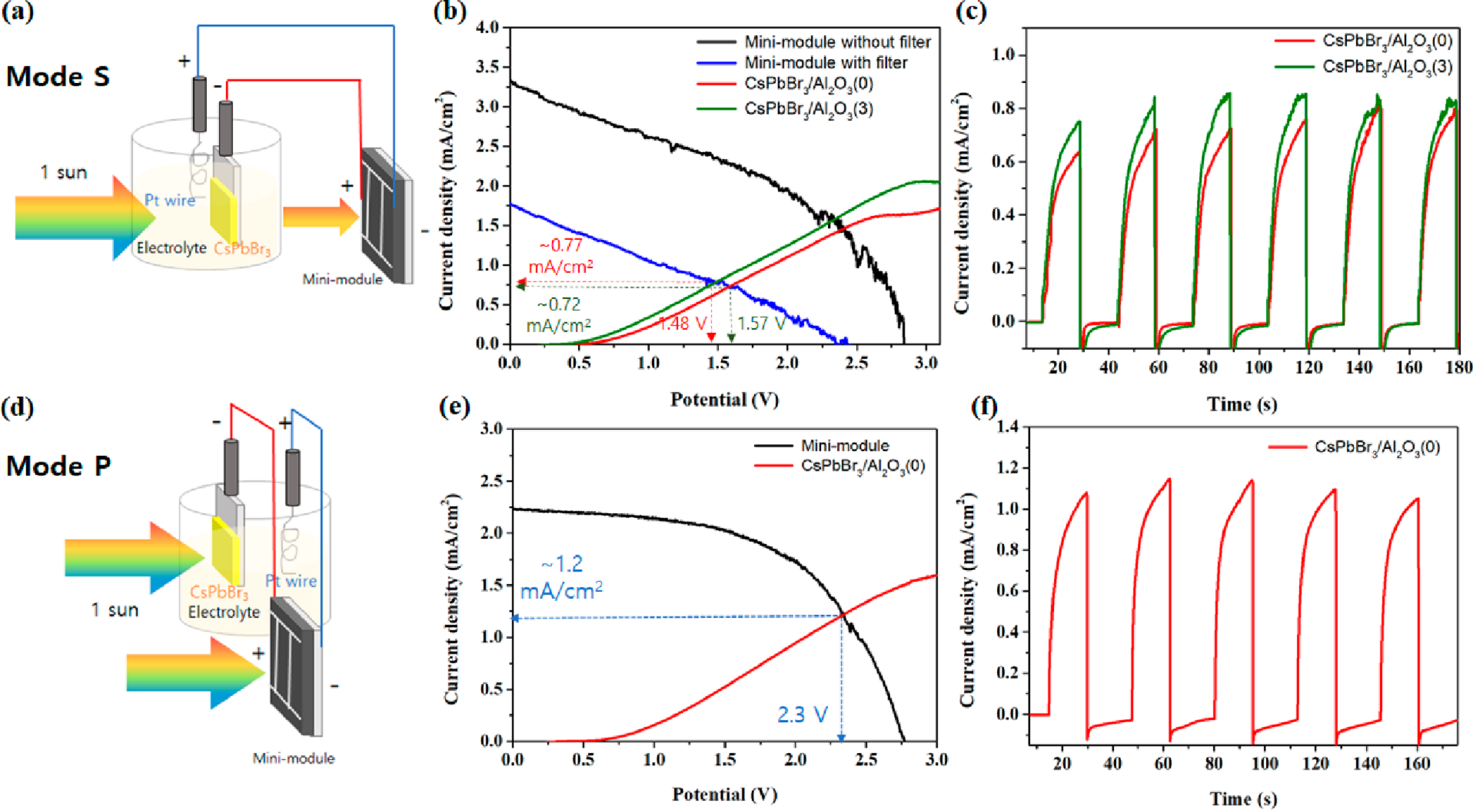
(a, d) Schematic diagrams of MAPbI_3_-based mini-module/CsPbBr_3_ PEC cell tandem devices for MBT oxidation with tandem serial and parallel illumination modes (modes S and P), respectively. (b, e) *J*–*V* curves of a mini-module and CsPbBr_3_-PEC cell to predict operating points in modes S and P, respectively. The *J*–*V* curves of the PEC cells were obtained in a two-electrode configuration, and the CsPbBr_3_ film was used as a filter for the mini-module to simulate mode S. (c, f) CA of the tandem device in modes S and P, respectively, without an external bias under chopped illumination. The light intensity for *J*–*V* and CA measurements was AM 1.5G (100 mW/cm^2^), and the illuminated areas of the mini-module were 1.0 and 2.4 cm^2^ in modes S and P, respectively.

In summary, a nanoscale CsPbBr_3_ perovskite photosensitizer can be formed in situ on the surface of meso-TiO_2_ film by a two-step spin-coating method using a low concentration of precursor solutions (<0.3 M). The meso-TiO_2_/nano-CsPbBr_3_ photoelectrode architecture is advantageous for the MBT oxidation reaction, since a higher density of catalytically active sites is available by increasing the contact area with the electrolyte and facilitating the electron transfer from CsPbBr_3_ to the meso-TiO_2_ film. Indeed, the PEC behavior of the meso-TiO_2_/nano-CsPbBr_3_ photoanode measured under various conditions confirmed that the photocurrent was obtained from the MBT oxidation by photoexcited CsPbBr_3_. Through the optimization process, a high photocurrent of 2.34 ± 0.08 mA/cm^2^ was obtained at 0.8 V (V vs Ag/Ag^+^) under standard AM 1.5G (100 mW/cm^2^) illumination. In addition, a thin layer of Al_2_O_3_ for the passivation effect was introduced on the surface of the meso-TiO_2_/nano-CsPbBr_3_ photoanode cell by the ALD method, and the highest photocurrent of 3.02 ± 0.03 mA/cm^2^ was obtained with 3 cycles of ALD. Consequently, the addition of perovskite boosts in most of the cases 1 or 2 magnitudes of the reaction current compared to previously reported PEC-degradation systems based on oxide electrodes for various organic pollutants, as shown in Table S4. This result looks promising for further enhancements by optimizing nanoscale interfaces, though the typical bulk-film-derived PEC currents are higher at the current stage as summarized in Table S5. Interestingly, the Al_2_O_3_-deposited electrode showed improved stability when it was stirred during the CA measurement. This is because stirring facilitated the supply of MBT to the surface of the meso-structured electrode, leading to a continued reaction. Furthermore, the meso-TiO_2_/nano-CsPbBr_3_ PEC cell and MAPbI_3_-based mini-module were combined for an unassisted MBT oxidation reaction driven directly by sunlight. The performance of this PV/PEC tandem device was evaluated in two different configurations, tandem serial and parallel illumination modes, yielding photocurrents of about 0.8 and 1.0 mA/cm^2^, respectively. The combination of such mini-modules and photosensitizer-based photoelectrodes opens up promising perspectives for the exploitation of halide perovskite based systems for several PEC reactions.

## Supporting Information Available

Experimental details of the photoelectrode preparation, Al_2_O_3_ ALD process, mini-module fabrication, characterizations, additional results and information including statistical photocurrents of PEC cells, LSVs for optimization of PEC cells, XPS, CA, absorbance spectra, and mini-module architecture (PDF)

## References

[ref1] Dahl S.; Chorkendorff I. Towards Practical Implementation. Nat. Mater. 2012, 11 (2), 100–101. 10.1038/nmat3233. 22270823

[ref2] National Renewable Energy Laboratory. https://www.nrel.gov/pv/assets/pdfs/best-research-cell-efficiencies.pdf. Downloaded July 18, 2023.

[ref3] Park S.; Chang W. J.; Lee C. W.; Park S.; Ahn H.-Y.; Nam K. T. Photocatalytic Hydrogen Generation from Hydriodic Acid Using Methylammonium Lead Iodide in Dynamic Equilibrium with Aqueous Solution. Nat. Energy 2017, 2 (1), 16185 10.1038/nenergy.2016.185.

[ref4] Xu Y.-F.; Yang M.-Z.; Chen B.-X.; Wang X.-D.; Chen H.-Y.; Kuang D.-B.; Su C.-Y. A CsPbBr_3_ Perovskite Quantum Dot/Graphene Oxide Composite for Photocatalytic CO_2_ Reduction. J. Am. Chem. Soc. 2017, 139 (16), 5660–5663. 10.1021/jacs.7b00489. 28385017

[ref5] Da P.; Cha M.; Sun L.; Wu Y.; Wang Z.-S.; Zheng G. High-Performance Perovskite Photoanode Enabled by Ni Passivation and Catalysis. Nano Lett. 2015, 15 (5), 3452–3457. 10.1021/acs.nanolett.5b00788. 25915528

[ref6] Singh S.; Chen H.; Shahrokhi S.; Wang L. P.; Lin C. H.; Hu L.; Guan X.; Tricoli A.; Xu Z. J.; Wu T. Hybrid Organic-Inorganic Materials and Composites for Photoelectrochemical Water Splitting. ACS Energy Lett. 2020, 5 (5), 1487–1497. 10.1021/acsenergylett.0c00327.

[ref7] Wang X.-D.; Huang Y.-H.; Liao J.-F.; Wei Z.-F.; Li W.-G.; Xu Y.-F.; Chen H.-Y.; Kuang D.-B. Surface Passivated Halide Perovskite Single-Crystal for Efficient Photoelectrochemical Synthesis of Dimethoxydihydrofuran. Nat. Commun. 2021, 12 (1), 1202 10.1038/s41467-021-21487-8. 33619252 PMC7900229

[ref8] Gualdrón-Reyes A. F.; Mesa C. A.; Giménez S.; Mora-Seró I. Application of Halide Perovskite Nanocrystals in Solar-Driven Photo(Electro)Catalysis. Solar RRL 2022, 6 (7), 2200012 10.1002/solr.202200012.

[ref9] DuBose J. T.; Kamat P. V. Efficacy of Perovskite Photocatalysis: Challenges to Overcome. ACS Energy Lett. 2022, 7 (6), 1994–2011. 10.1021/acsenergylett.2c00765.

[ref10] Han H. S.; Shin S.; Kim D. H.; Park I. J.; Kim J. S.; Huang P.-S.; Lee J.-K.; Cho I. S.; Zheng X. Boosting the Solar Water Oxidation Performance of a BiVO_4_ Photoanode by Crystallographic Orientation Control. Energy Environ. Sci. 2018, 11 (5), 1299–1306. 10.1039/C8EE00125A.

[ref11] Lee D. K.; Choi K.-S. Enhancing Long-Term Photostability of BiVO_4_ Photoanodes for Solar Water Splitting by Tuning Electrolyte Composition. Nat. Energy 2018, 3 (1), 53–60. 10.1038/s41560-017-0057-0.

[ref12] Kim T. W.; Ping Y.; Galli G. A.; Choi K.-S. Simultaneous Enhancements in Photon Absorption and Charge Transport of Bismuth Vanadate Photoanodes for Solar Water Splitting. Nat. Commun. 2015, 6 (1), 8769 10.1038/ncomms9769. 26498984 PMC4640143

[ref13] Kim J. H.; Jang J.-W.; Jo Y. H.; Abdi F. F.; Lee Y. H.; van de Krol R.; Lee J. S. Hetero-Type Dual Photoanodes for Unbiased Solar Water Splitting with Extended Light Harvesting. Nat. Commun. 2016, 7 (1), 13380 10.1038/ncomms13380. 27966548 PMC5477502

[ref14] Pulignani C.; Mesa C. A.; Hillman S. A. J.; Uekert T.; Giménez S.; Durrant J. R.; Reisner E. Rational Design of Carbon Nitride Photoelectrodes with High Activity Toward Organic Oxidations. Angew. Chem., Int. Ed. 2022, 61 (50), 202211587 10.1002/anie.202211587. PMC10099510 36224107

[ref15] Zhang L.; Liardet L.; Luo J.; Ren D.; Grätzel M.; Hu X. Photoelectrocatalytic Arene C–H Amination. Nat. Catal. 2019, 2 (4), 366–373. 10.1038/s41929-019-0231-9. PMC6459354 30984910

[ref16] Chen J.; Yin J.; Zheng X.; Ait Ahsaine H.; Zhou Y.; Dong C.; Mohammed O. F.; Takanabe K.; Bakr O. M. Compositionally Screened Eutectic Catalytic Coatings on Halide Perovskite Photocathodes for Photoassisted Selective CO_2_ Reduction. ACS Energy Lett. 2019, 4 (6), 1279–1286. 10.1021/acsenergylett.9b00751.

[ref17] Song J.; Li J.; Li X.; Xu L.; Dong Y.; Zeng H. Quantum Dot Light-Emitting Diodes Based on Inorganic Perovskite Cesium Lead Halides (CsPbX_3_). Adv. Mater. 2015, 27 (44), 7162–7167. 10.1002/adma.201502567. 26444873

[ref18] Cardenas-Morcoso D.; Gualdrón-Reyes A. F.; Ferreira Vitoreti A. B.; García-Tecedor M.; Yoon S. J.; Solis De La Fuente M.; Mora-Seró I.; Gimenez S. Photocatalytic and Photoelectrochemical Degradation of Organic Compounds with All-Inorganic Metal Halide Perovskite Quantum Dots. J. Phys. Chem. Lett. 2019, 10 (3), 630–636. 10.1021/acs.jpclett.8b03849. 30673244

[ref19] Clarke B. O.; Smith S. R. Review of ‘Emerging’ Organic Contaminants in Biosolids and Assessment of International Research Priorities for the Agricultural Use of Biosolids. Environ. Int. 2011, 37 (1), 226–247. 10.1016/j.envint.2010.06.004. 20797791

[ref20] De Wever H.; Verachtert H. Biodegradation and toxicity of benzothiazoles. Wat. Res. 1997, 31 (11), 2673–2684. 10.1016/S0043-1354(97)00138-3.

[ref21] Sorahan T. Cancer Risks in Chemical Production Workers Exposed to 2-Mercaptobenzothiazole. Occup Environ. Med. 2008, 66 (4), 269–273. 10.1136/oem.2008.041400. 19158128

[ref22] Hagfeldt A.; Boschloo G.; Sun L.; Kloo L.; Pettersson H. Dye-Sensitized Solar Cells. Chem. Rev. 2010, 110 (11), 6595–6663. 10.1021/cr900356p. 20831177

[ref23] Pan Z.; Rao H.; Mora-Seró I.; Bisquert J.; Zhong X. Quantum Dot-Sensitized Solar Cells. Chem. Soc. Rev. 2018, 47 (20), 7659–7702. 10.1039/C8CS00431E. 30209490

[ref24] Chebrolu V. T.; Kim H.-J. Recent Progress in Quantum Dot Sensitized Solar Cells: An Inclusive Review of Photoanode, Sensitizer, Electrolyte, and the Counter Electrode. J. Mater. Chem. C 2019, 7 (17), 4911–4933. 10.1039/C8TC06476H.

[ref25] Liu F.; Zhang Y.; Ding C.; Toyoda T.; Ogomi Y.; Ripolles T. S.; Hayase S.; Minemoto T.; Yoshino K.; Dai S.; Shen Q. Ultrafast Electron Injection from Photoexcited Perovskite CsPbI_3_ QDs into TiO_2_ Nanoparticles with Injection Efficiency near 99%. J. Phys. Chem. Lett. 2018, 9 (2), 294–297. 10.1021/acs.jpclett.7b03062. 29286666

[ref26] Lee H. J.; Cho K. T.; Paek S.; Lee Y.; Huckaba A. J.; Queloz V. I. E.; Zimmermann I.; Grancini G.; Oveisi E.; Yoo S. M.; Lee S. Y.; Shin T.; Kim M.; Nazeeruddin M. K. A Facile Preparative Route of Nanoscale Perovskites over Mesoporous Metal Oxide Films and Their Applications to Photosensitizers and Light Emitters. Adv. Funct. Mater. 2018, 28 (39), 1803801 10.1002/adfm.201803801.

[ref27] Kim M.; Lee S. Y.; Yoo S. M.; Paek S.; Lee Y.; Cho K. T.; Zimmermann I.; Kim H. Y.; Kim B. S.; Song M. K.; Shin T.; Kim K.; Huckaba A. J.; Nazeeruddin M. K.; Lee H. J. Effective Preparation of Nanoscale CH_3_NH_3_PbI_3_ Perovskite Photosensitizers for Mesoporous TiO_2_-Based Solar Cells by Successive Precursor Layer Adsorption and Reaction Process. Energy Technol. 2020, 8 (4), 1901186 10.1002/ente.201901186.

[ref28] Yoo S. M.; Lee S. Y.; Velilla Hernandez E.; Kim M.; Kim G.; Shin T.; Nazeeruddin M. K.; Mora-Seró I.; Lee H. J. Nanoscale Perovskite-Sensitized Solar Cell Revisited: Dye-Cell or Perovskite-Cell?. ChemSusChem 2020, 13 (10), 2571–2576. 10.1002/cssc.202000223. 32202374 PMC7496478

[ref29] Yoo S. M.; Yoon S. J.; Anta J. A.; Lee H. J.; Boix P. P.; Mora-Seró I. An Equivalent Circuit for Perovskite Solar Cell Bridging Sensitized to Thin Film Architectures. Joule 2019, 3 (10), 2535–2549. 10.1016/j.joule.2019.07.014.

[ref30] Roelofs K. E.; Brennan T. P.; Dominguez J. C.; Bailie C. D.; Margulis G. Y.; Hoke E. T.; McGehee M. D.; Bent S. F. Effect of Al_2_O_3_ Recombination Barrier Layers Deposited by Atomic Layer Deposition in Solid-State CdS Quantum Dot-Sensitized Solar Cells. J. Phys. Chem. C 2013, 117 (11), 5584–5592. 10.1021/jp311846r.

[ref31] Loiudice A.; Saris S.; Oveisi E.; Alexander D. T. L.; Buonsanti R. CsPbBr_3_ QD/AlO_x_ Inorganic Nanocomposites with Exceptional Stability in Water, Light, and Heat. Angew. Chem., Int. Ed. 2017, 56 (36), 10696–10701. 10.1002/anie.201703703. 28547826

[ref32] Zhang K.; Ma M.; Li P.; Wang D. H.; Park J. H. Water Splitting Progress in Tandem Devices: Moving Photolysis beyond Electrolysis. Adv. Energy Mater. 2016, 6 (15), 1600602 10.1002/aenm.201600602.

[ref33] Chen Y. S.; Manser J. S.; Kamat P. V. All Solution-Processed Lead Halide Perovskite-BiVO_4_ Tandem Assembly for Photolytic Solar Fuels Production. J. Am. Chem. Soc. 2015, 137 (2), 974–981. 10.1021/ja511739y. 25543877

[ref34] Gurudayal; Sabba D.; Kumar M. H.; Wong L. H.; Barber J.; Grätzel M.; Mathews N. Perovskite-Hematite Tandem Cells for Efficient Overall Solar Driven Water Splitting. Nano Lett. 2015, 15 (6), 3833–3839. 10.1021/acs.nanolett.5b00616. 25942281

[ref35] Yoo S. M.; Lee S. Y.; Kim G.; Hernandez E. V.; Mora-Seró I.; Yoon S. J.; Shin T.; Lee S. H.; Ahn S.; Song M. K.; Kim M.; Lee H. J. Preparation of Nanoscale Inorganic CsPbI_x_Br_3-x_ Perovskite Photosensitizers on the Surface of Mesoporous TiO_2_ Film for Solid-State Sensitized Solar Cells. Appl. Surf. Sci. 2021, 551, 149387 10.1016/j.apsusc.2021.149387.

[ref36] Zhang H.; Mao J.; He H.; Zhang D.; Zhu H. L.; Xie F.; Wong K. S.; Grätzel M.; Choy W. C. H. A Smooth CH_3_NH_3_PbI_3_ Film via a New Approach for Forming the PbI_2_ Nanostructure Together with Strategically High CH_3_NH_3_I Concentration for High Efficient Planar-Heterojunction Solar Cells. Adv. Energy Mater. 2015, 5 (23), 1501354 10.1002/aenm.201501354.

[ref37] Shi Y.; Wang X.; Zhang H.; Li B.; Lu H.; Ma T.; Hao C. Effects of 4-*tert*-butylpyridine on Perovskite Formation and Performance of Solution-Processed Perovskite Solar Cells. J. Mater. Chem. A 2015, 3 (44), 22191–22198. 10.1039/C5TA05988G.

[ref38] Shahrokhian S.; Amini M. K.; Mohammadpoor-Baltork I.; Tangestaninejad S. Potentiometric Detection of 2-Mercaptobenzimidazole and 2-Mercaptobenzothiazole at Cobalt Phthalocyanine Modified Carbon-Paste Electrode. Electroanalysis 2000, 12 (11), 863–867. 10.1002/1521-4109(200007)12:11<863::AID-ELAN863>3.0.CO;2-H.

[ref39] Abdi F. F.; Savenije T. J.; May M. M.; Dam B.; Van De Krol R. The Origin of Slow Carrier Transport in BiVO_4_ Thin Film Photoanodes: A Time-Resolved Microwave Conductivity Study. J. Phys. Chem. Lett. 2013, 4 (16), 2752–2757. 10.1021/jz4013257.

[ref40] Seabold J. A.; Choi K. S. Efficient and Stable Photo-Oxidation of Water by a Bismuth Vanadate Photoanode Coupled with an Iron Oxyhydroxide Oxygen Evolution Catalyst. J. Am. Chem. Soc. 2012, 134 (4), 2186–2192. 10.1021/ja209001d. 22263661

[ref41] Xiao S.; Chen H.; Yang Z.; Long X.; Wang Z.; Zhu Z.; Qu Y.; Yang S. Origin of the Different Photoelectrochemical Performance of Mesoporous BiVO_4_ Photoanodes between the BiVO_4_ and the FTO Side Illumination. J. Phys. Chem. C 2015, 119 (41), 23350–23357. 10.1021/acs.jpcc.5b07505.

[ref42] Bisquert J.; Vikhrenko V. S. Interpretation of the Time Constants Measured by Kinetic Techniques in Nanostructured Semiconductor Electrodes and Dye-Sensitized Solar Cells. J. Phys. Chem. B 2004, 108 (7), 2313–2322. 10.1021/jp035395y.

[ref43] Liu D.; Hu Z.; Hu W.; Wangyang P.; Yu K.; Wen M.; Zu Z.; Liu J.; Wang M.; Chen W.; Zhou M.; Tang X.; Zang Z. Two-Step Method for Preparing All-Inorganic CsPbBr_3_ Perovskite Film and Its Photoelectric Detection Application. Mater. Lett. 2017, 186, 243–246. 10.1016/j.matlet.2016.10.015.

[ref44] Duan J.; Zhao Y.; He B.; Tang Q. Simplified Perovskite Solar Cell with 4.1% Efficiency Employing Inorganic CsPbBr_3_ as Light Absorber. Small 2018, 14 (20), 1704443 10.1002/smll.201704443. 29665218

[ref45] Ma Y.; Zhao Q. A Strategic Review on Processing Routes towards Scalable Fabrication of Perovskite Solar Cells. J. Energy Chem. 2022, 64, 538–560. 10.1016/j.jechem.2021.05.019.

[ref46] Wang H.; Qin Z.; Miao Y.; Zhao Y. Recent Progress in Large-Area Perovskite Photovoltaic Modules. Trans. Tianjin Univ. 2022, 28, 323–340. 10.1007/s12209-022-00341-y.

[ref47] Di Giacomo F.; Castriotta L. A.; Kosasih F. U.; Di Girolamo D.; Ducati C.; Di Carlo A. Upscaling Inverted Perovskite Solar Cells: Optimization of Laser Scribing for Highly Efficient Mini-Modules. Micromachines 2020, 11 (12), 1127 10.3390/mi11121127. 33419276 PMC7767295

[ref48] Li D.; Zhang D.; Lim K. S.; Hu Y.; Rong Y.; Mei A.; Park N. G.; Han H. A Review on Scaling Up Perovskite Solar Cells. Adv. Funct. Mater. 2021, 31 (12), 2008621 10.1002/adfm.202008621.

[ref49] Bu T.; Liu X.; Li J.; Huang W.; Wu Z.; Huang F.; Cheng Y. B.; Zhong J. Dynamic Antisolvent Engineering for Spin Coating of 10 × 10 cm^2^ Perovskite Solar Module Approaching 18. Solar RRL 2020, 4 (2), 1900263 10.1002/solr.201900263.

[ref50] Ding C.; Qin W.; Wang N.; Liu G.; Wang Z.; Yan P.; Shi J.; Li C. Solar-to-Hydrogen Efficiency Exceeding 2.5% Achieved for Overall Water Splitting with an All Earth-Abundant Dual-Photoelectrode. Phys. Chem. Chem. Phys. 2014, 16 (29), 15608–15614. 10.1039/C4CP02391A. 24956231

