# Supplementary material for: A Perovskite Photovoltaic Mini-Module-CsPbBr3 Photoelectrochemical Cell Tandem Device for Solar-Driven Degradation of Organic Compounds
Source: ACS Energy Lett. 2023 Oct 2;8(10):4488–95. doi: 10.1021/acsenergylett.3c01361 (PMC10580309; doi:10.1021/acsenergylett.3c01361)
Supplement: Supplementary file 1 — nz3c01361_si_001.pdf [file nz3c01361_si_001.pdf]

A Perovskite Photovoltaic Mini-Module-$CsPbBr_3$ Photoelectrochemical Cell Tandem Device for Solar Driven Degradation of Organic Compounds

Seul-Yi Lee[1,2], Patricio Serafini[2], Sofia Masi[2], Andrés F. Gualdrón-Reyes[2,3], Camilo A. Mesa[2], Jhonatan Rodríguez-Pereira[4,5], Sixto Giménez[2], Hyo Joong Lee[1]*, and Iván Mora-Seró[2]**

[1]Department of Chemistry and Research Institute of Physics & Chemistry, Jeonbuk National University, Jeonju 561-756, South Korea

[2] Institute of Advanced Materials, Universitat Jaume I, Castelló de la Plana 12071, Spain.

[3]Facultad de Ciencias, Instituto de Ciencias Químicas, Isla Teja, Universidad Austral de Chile, Valdivia 5090000, Chile.

[4]Center of Materials and Nanotechnologies, Faculty of Chemical Technology, University of Pardubice, Nam. Cs. Legii 565, Pardubice 53002, Czech Republic.

[5]Central European Institute of Technology, Brno University of Technology, Purkynova 123, Brno 61200, Czech Republic.

Corresponding Author: sjulia@uji.es , solarlee@jbnu.ac.kr, sero@uji.es

Experimental section

The preparation of the FTO/meso-TiO$_2$/nano-CsPbBr$_3$ PEC electrode: Fluorine-doped tin oxide-coated (FTO) glasses (1.0 × 3.0 cm^2, Pilkington TEC15, ~15 Ω/cm) were cleaned with soap water solution and sonicated sequentially in acetone and 2-propanol solvents for 10 min each. After that, the substrates were dried with air gun and completed with a UV-O$_3$ treatment for 15 min. For the compact TiO$_2$ layer, 0.2 M Ti-precursor solution was prepared by diluting titanium diisopropoxidebis(acetylacetonate) in 1-butanol (Sigma-Aldrich, 99.8%) and spin coated with 100 μL of the solution at 2,000 rpm for 30 s. The films were heated slowly up to 500℃ and kept for another 30 min. The meso-TiO$_2$ layer was spin-coated using diluted TiO$_2$ paste (a 1.0 g of 30NR-D TiO$_2$ paste from great cell was dissolved in 3.0 ml, 2.5 ml and 2.0 ml of ethanol, respectively)

at 1,000 rpm for 25 s and annealed at 500℃ for 30 min. The $CsPbBr_3$ perovskite nanoparticles were formed on the surface of TiO_2 particulate films directly using a two-step spin-coating method as shown in **Figure 1**a. For the first step, lead-precursor solution was prepared by mixing 0.3 M lead(Ⅱ) bromide ($PbBr_2$, TCI, 98.0%) and 0.3 M 4-*tert*-butylpyridine (tBP, Sigma-Aldrich, 98%) in N,N-dimethylformamide (DMF, Sigma-Aldrich, 99.8%) and then 100 μL of the solution was dropped over the FTO/meso-TiO_2 electrode and spin coated at 4,000 rpm with 400 rpm/s acceleration for 40 s and dried at 70℃ for 5 min. For the second step, 0.03 M cesium bromide (CsBr, Sigma-Aldrich, 99.999%) was dissolved in methanol (Sigma-Aldrich, 99.8%) and 500 μL of the CsBr solution was dropped on the $PbBr_2$-deposited electrode while the electrode was spun at 2000 rpm. Then, the film was annealed at 280℃ for 3 min. All deposition processes were carried out in the air, and the active area was 1.0 cm^2.

Atomic layer deposition of aluminum oxide (Al_2O_3): The Al_2O_3 thin layer was deposited on the meso-TiO_2/nano-$CsPbBr_3$ coated electrode by a commercial atomic layer deposition (ALD) machine (AT-410, Anric Technologies) with trimethylaluminum (TMA) and water (H_2O) as the precursors. The precursors were maintained at room temperature and carried to a preheated (150℃) reaction chamber with a constant N_2 flow of 29 sccm. For each ALD cycle, the process consisted

of 3 pulses of TMA, 11 s of N_2 purge, 2 pulses of H_2O and 13 s of N_2 purge was conducted. A 0.91 Å thick Al_2O_3 layer was deposited for each cycle. The highest performance was obtained with 3 cycles.

The fabrication of $MAPbI_3$-based minimodules: The mini-module was fabricated with the architecture observed in **Figure S7**. The FTO glass (2.5× 2.5 cm^2, Pilkington TEC15, ~15 Ω/cm) was patterned with a glasscutter to remove the FTO coating for the P1 connection. Then, the patterned substrate was cleaned with deionized water, acetone, and ethanol in an ultrasonic cleaner for 15 min for each solvent. After being dried by air flow, the substrate was put in an UV-O_3 for 15 min to remove organic residues. For the electron transport layer, SnO_2 was deposited by spin-coating the dissolution of SnO_2 3% from colloid precursor (Alfa Aesar, 15% in H_2O colloidal dispersion) at 3,000 rpm for 30 s, and the film was heated at 150°C for 30 min. The electrode was exposed to UV-O_3 for 20 min before $MAPbI_3$ perovskite deposition. The $MAPbI_3$ perovskite solution was prepared by weighting methylammonium iodide (MAI, Greatcellsolar, 98%) and lead iodide (PbI_2, TCI, 98%) at 1.4 M concentration and dissolving the precursors in 4:1 DMF:DMSO (v;v). To deposit the perovskite on the substrate, 80 μL of the perovskite solution was spin-coated at 4,000 rpm for 40 s, and then annealed the perovskite film at 130°C for 10 min. For hole

transporting layer, a spiro-OMeTAD solution was prepared by diluting spiro-OMeTAD (Feiming chemical limited, 99%) in chlorobenzene (CB, Sigma-Aldrich, 99.8%) (85.5 mg/mL) doped with 28.8 μL of tBP and 17.8 μL of lithium bis(trifluoromethylsulfonyl)imide (Li-TFSI, Sigma-Aldrich, 99.95%) stock solution (the stock solution was made by dissolving 520 mg of Li-TFSI in 1mL of acetonitrile (Sigma-Aldrich, 99.8%)), and spin-coated at 4,000 rpm for 20 s onto the top annealed perovskite layer. After that, the P2 connection was performed with enough power to avoid the FTO suppression. Finally, Au electrode with a thickness of 80 nm was deposited by thermal evaporation and the P3 connection was conducted manually using a cutter. The total active area was 2.4 cm^2.

Photoelectrochemical characterization of $CsPbBr_3$-based photoanode: To optimize the $CsPbBr_3$-based photoanode electrode, linear sweep voltammetry (LSV) and chronoamperometry (CA) measurements were conducted in various conditions with a three-electrode configuration using an Autolab Potentiostat/Galvanostat. A non-aqueous Ag/Ag^+ electrode (ALS, Japan) and a platinum (Pt) wire were used as reference and counter electrode, respectively. The meso-TiO_2/nano-$CsPbBr_3$-based photoanode with/without Al_2O_3 ALD was used as the working electrode. The supporting electrolyte was prepared by dissolving 0.1 M tetrabutylammonium hexafluorophosphate (Bu_4NPF_6, Sigma-Aldrich, 99%) in dichloromethane (DCM, Sigma-Aldrich,

99.8%). The desired concentration of 2-mercaptobenzothiazole (MBT, Sigma-Aldrich, 97%) was added to supporting electrolyte, but the concentration of MBT was 0.05 M unless otherwise specified. Electrodes were front-side illuminated with a Xe lamp (Oriel 300W) at standard 1 sun (100 mW/cm^2) except for the experiment comparing front and back side illumination. The scan rate was 10 mV/s.

Current density-potential (J-V) curves of $MAPbI_3$-based mini-module and $CsPbBr_3$-based photoanode: J-V curves of both devices were obtained using the same potentiostat (Autolab Potentiostat/Galvanostat) under standard AM 1.5G illumination simulated with a Xe lamp (Oriel 300W). For the mini-module, J-V curves were measured in two different ways depending on the configuration of tandem device. In the case of mode S (**Figure 4**a), meso-TiO_2/nano-$CsPbBr_3$ film was placed in front of the mini-module to simulate mode S, and then a mask which has the same active area (1.0 cm^2) with meso-TiO_2/nano-$CsPbBr_3$ PEC cell was also put between the meso-TiO_2/nano-$CsPbBr_3$ film and mini-module. To maintain the high voltage of the mini-module, the mask had to be positioned so that light could be irradiated on all three solar cells of the mini-module. In order to compare the performance of mini-module with/without $CsPbBr_3$ filter, the J-V curve of mini-module without the filter was also obtained with the mask. In the case of mode P

(**Figure 4d**), J-V curve of mini-module was collected without mask and $CsPbBr_3$ filter because light of the same intensity was incident on both devices. The active areas of $CsPbBr_3$ photoanode and mini-module were 1.0 and 2.4 cm^2, respectively. For J-V curves of $CsPbBr_3$-based photoanode with/without 3 cycles of Al_2O_3 ALD, the electrode was front-side illuminated, and the measurements were carried out in the electrolyte of 0.1 M Bu_4NPF_6 and 0.05 M MBT in DCM with a two-electrode configuration using Pt wire as a counter electrode. The scan rate of both devices was 10 mV/s.

Photoelectrochemical measurements in the tandem device: CA measurements of tandem device were done in two configurations with the same potentiostat and Xe lamp as the ones used for measuring the J-V curves. However, the connection between $CsPbBr_3$ PEC system and mini-module was the same regardless of the illumination mode. The $CsPbBr_3$-based photoanode was connected to the (+) of mini-module, and the (-) of mini-module was connected to Pt wire of PEC system through the potentiostat. Photocurrent was recorded under chopped standard AM 1.5G 1 sun illumination. For mode S, CA was measured by positioning the $CsPbBr_3$-based photoanode, mask, and mini-module in a straight line so that light could pass through them as shown in **Figure**

4a. In the case of mode P, CA was obtained by placing the PEC cell and mini-module parallel to the light without mask as shown in **Figure 4**d.

General characterization: The morphology of the photoanode films and the thickness of meso-TiO_2 films were analyzed with scanning electron microscopy (SEM, JEOL JSM7001F). The field emission transmission electron microscopy (FE-TEM) image was obtained with a Hitachi HF-3300. The absorbance and transmittance of $CsPbBr_3/Al_2O_3$(0) and $CsPbBr_3/Al_2O_3$(3) were measured on a Varian Cary 300 Bio spectrophotometer. Absorbance measurement was also conducted to check the degradation of MBT after stability test with/without stirring. For this, the initial 0.05 M MBT was diluted to 0.05 mM with the solution of 0.1 M Bu_4NPF_6 in DCM, and the electrolytes taken after the stability test were also diluted in the same way. X-ray diffraction (XRD) patterns of samples were obtained on a Bruker-AXS D4 Endeavor diffractometer with Cu K_α radiation source (λ = 1.54056 Å). The surface chemical state of the samples was estimated by X-ray photoelectron spectroscopy (XPS, ESCA-2R, Scienta-Omicron) using monochromatic Al Kα (1486.6 eV) radiation. The binding energy scale was referenced to adventitious carbon (284.8 eV). CasaXPS processing software (Casa software Ltd) was used to analyze the data.

Table S1. The photocurrent density of the meso-TiO_2/nano-$CsPbBr_3$ electrodes as a function of the concentration of MBT in the electrolyte at 0.8 V (V *vs.* Ag/Ag^+).

From now on, all the summarized photocurrent in the tables were obtained using three electrodes for each condition to ensure reproducibility.

**Sample**	**0.00 M (mA/cm^2)**	**0.03 M (mA/cm^2)**	**0.05 M (mA/cm^2)**	**0.07 M (mA/cm^2)**
1	0.33	2.11	2.40	1.79
2	0.21	2.03	2.23	1.61
3	0.21	1.90	2.38	1.78
Average	0.25±0.06	2.01±0.09	2.34±0.08	1.73±0.08

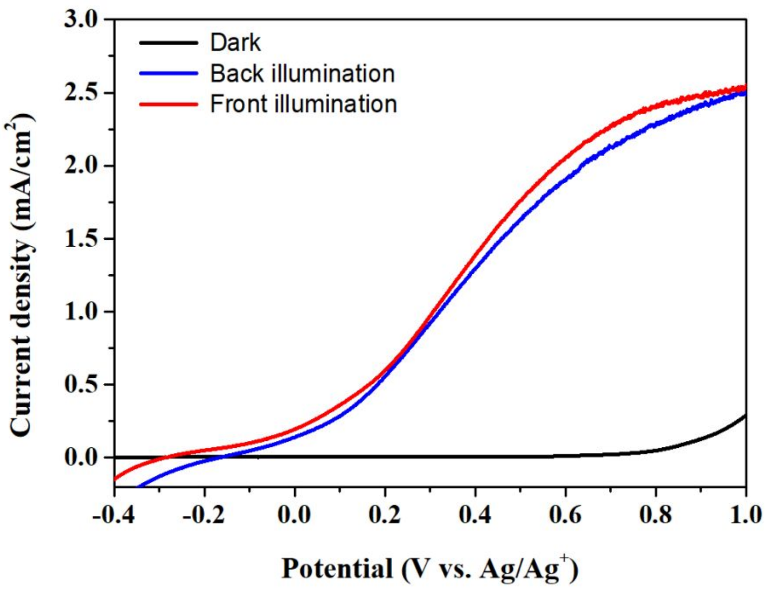

Figure S1. LSV of meso-TiO_2/nano-$CsPbBr_3$ electrode depending on front and back side illumination.

Table S2. The photocurrent density of the meso-TiO_2/nano-$CsPbBr_3$ electrodes comparing front and back side illumination at 0.8 V (V *vs.* Ag/Ag^+).

**Sample**	**Front illumination (mA/cm^2)**	**Back illumination (mA/cm^2)**
1	2.40	2.07
2	2.23	2.28
3	2.38	1.89
Average	2.34±0.08	2.08±0.16

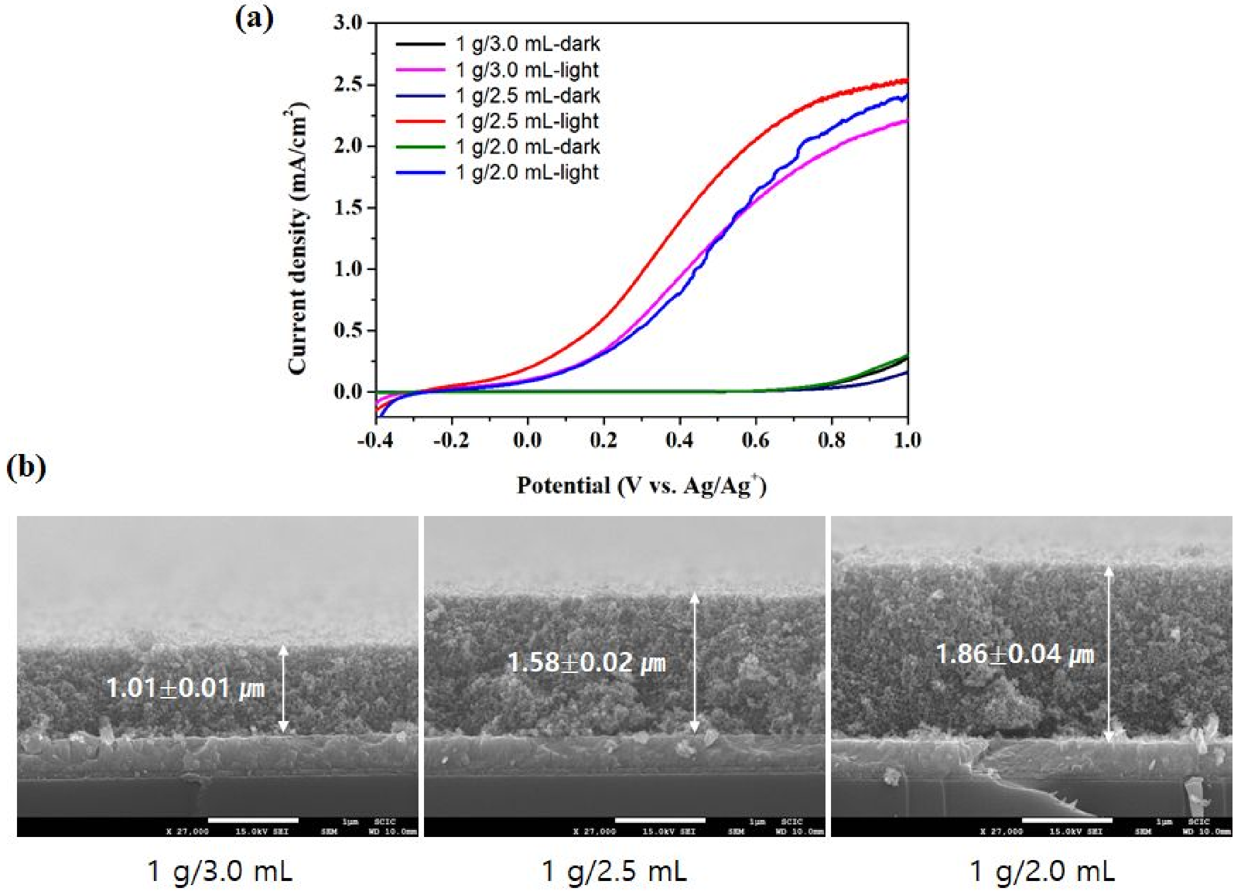

Figure S2. (a) LSV of meso-TiO_2/nano-$CsPbBr_3$ electrodes prepared with various TiO_2 pastes diluted in different volumes of ethanol. (b) The thickness of meso-TiO_2 film depending on the diluted TiO_2 pastes in different volumes of ethanol.

Table S3. The photocurrent density of the meso-TiO_2/nano-$CsPbBr_3$ electrodes with different cycles of Al_2O_3 ALD at 0.8 V (V *vs.* Ag/Ag^+).

Sample	0 cycle (mA/cm^2)	1 cycle (mA/cm^2)	2 cycles (mA/cm^2)	3 cycles (mA/cm^2)	4 cycles (mA/cm^2)
1	2.40	2.64	2.83	3.06	2.79
2	2.23	2.44	2.85	3.01	2.70
3	2.38	2.56	3.02	3.00	2.65
Average	2.34±0.08	2.55±0.08	2.90±0.09	3.02±0.03	2.71±0.06

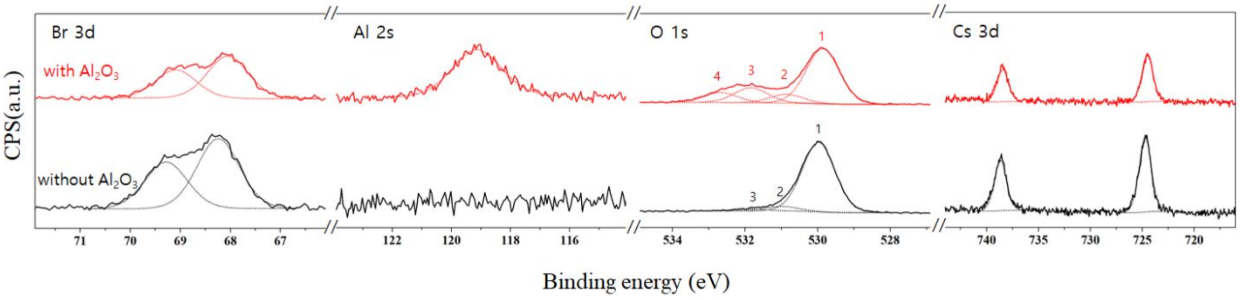

Figure S3. XPS analysis of the $CsPbBr_3$/Al_2O_3(0) (black) and $CsPbBr_3$/Al_2O_3(3) (red) films. The dashed lines show the fitted curves for each element.

The XPS spectra of Al 2s were reported instead of the Al 2p due to its overlapping with Cs 4d peaks. The O 1s core levels were fitted with several peaks: Peaks 1 and 2 could be from Ti-O and Al-O, respectively. Peak 3 could be related to the environmental conditions during the measurement. And peak 4 could be observed by oxygen bounded with materials used in the Al_2O_3 ALD process. The clear peaks 2 and 4 were observed for $CsPbBr_3/Al_2O_3(3)$ film but not for $CsPbBr_3/Al_2O_3(0)$.

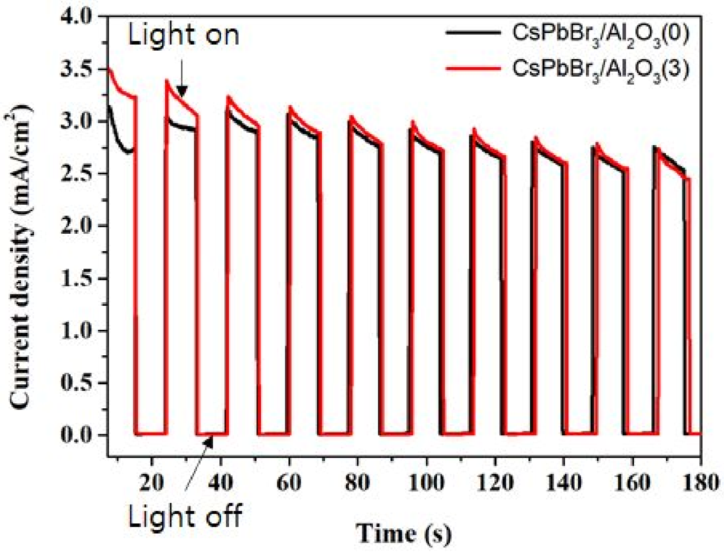

Figure S4. CA of the $CsPbBr_3/Al_2O_3(0)$ and $CsPbBr_3/Al_2O_3(3)$ photoanodes at 0.8 V (V *vs.* Ag/Ag^+) under chopped AM 1.5G (100 mW/cm^2) illumination.

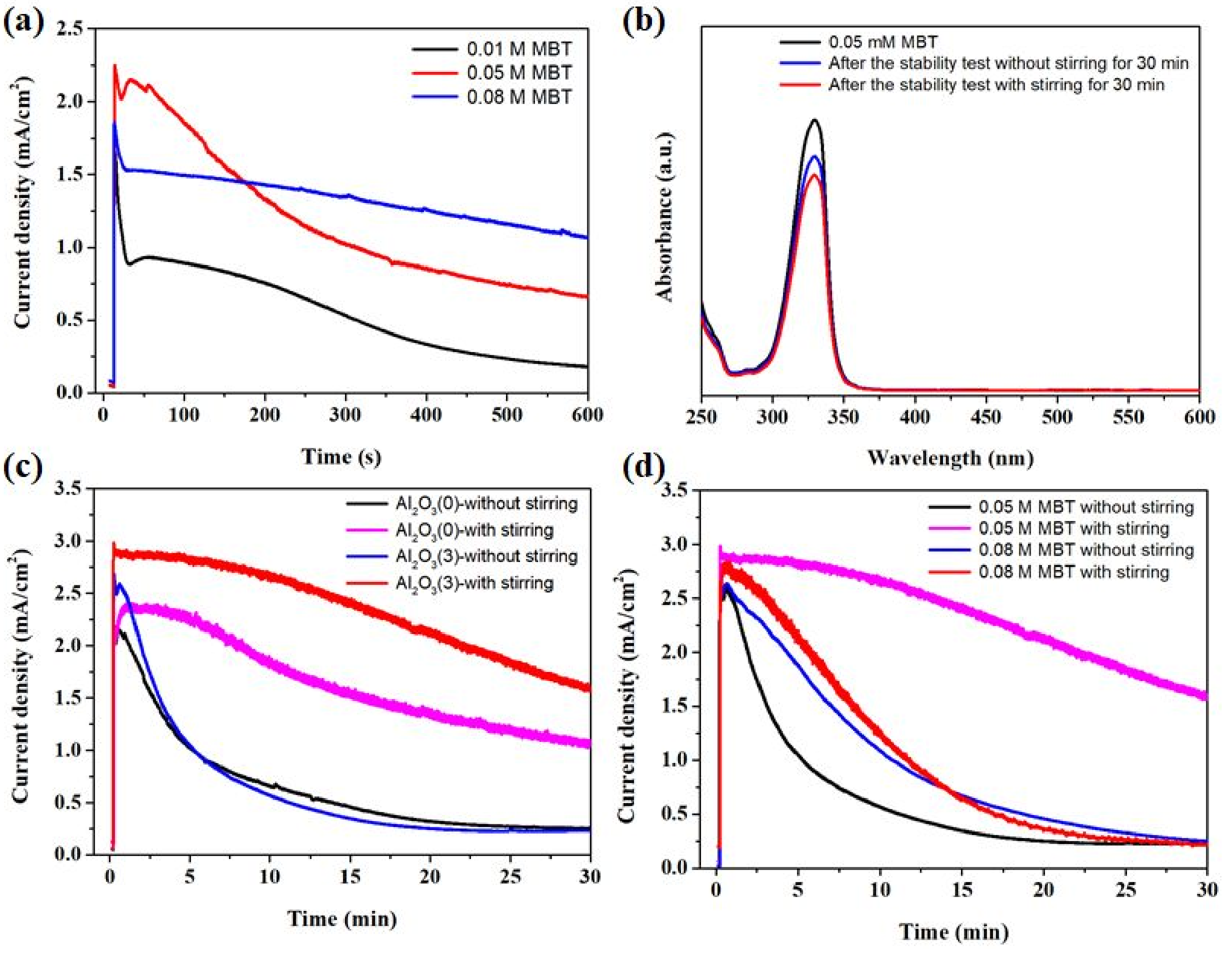

Figure S5. (a) Current density of meso-TiO_2/nano-$CsPbBr_3$ electrodes in different concentrations of MBT in the electrolyte, measured without stirring. (b) Absorbance spectra of diluted electrolyte

with and without stirring during the stability test for 30 min (the initial MBT concentration of 0.05 M in the electrolyte was diluted to 0.05 mM, and the electrolytes after the stability test were diluted in the same scale). (c) The stability test of **Figure 3**d for extended time to 30 min. (d) CA with and without stirring during the stability test of $CsPbBr_3/Al_2O_3$(3) electrode in the electrolyte including 0.05 M or 0.08 M MBT. The CA measurements were conducted at 0.8 V (V *vs.* Ag/Ag^+) under standard AM 1.5G irradiation.

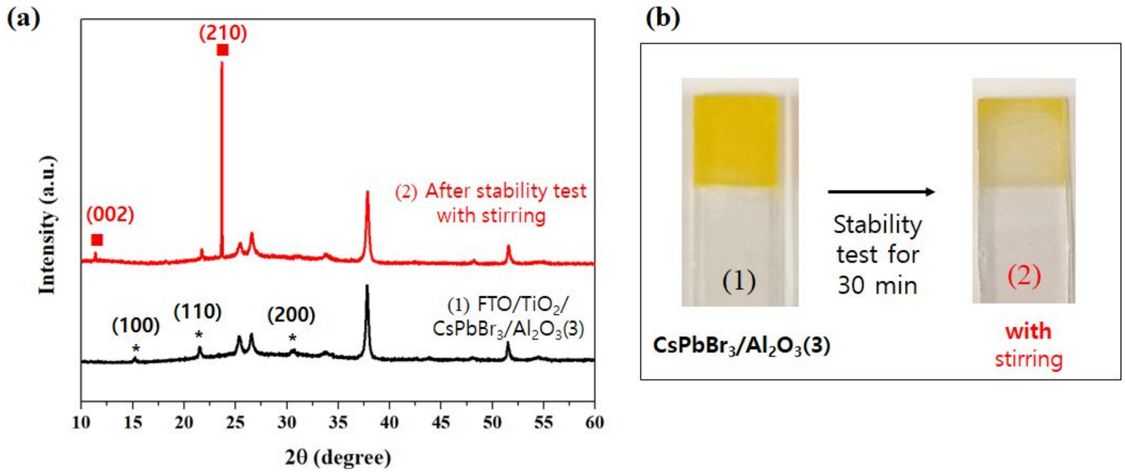

Figure S6. (a) XRD pattern change of $FTO/TiO_2/CsPbBr_3/Al_2O_3$ photoanode after the stability test with stirring for 30 min in the electrolyte containing 0.05 M MBT [asterisk marks in the lower part (1) show the main peak of $CsPbBr_3$ XRD data before the stability test while some peaks marked with red squares are from $CsPb_2Br_5$[1] in the upper part (2) after the stability test] and (b) photos of electrodes used before and after stability test.

[1] Tang, X.; Han, S.; Zu, Z.; Hu, W.; Zhou, D.; Du, J.; Hu, Z.; Li, S.; Zang, Z. All-Inorganic Perovskite $CsPb_2Br_5$ Microsheets for Photodetector Application. *Front. Phys.* **2018**, *5* (1), 69.

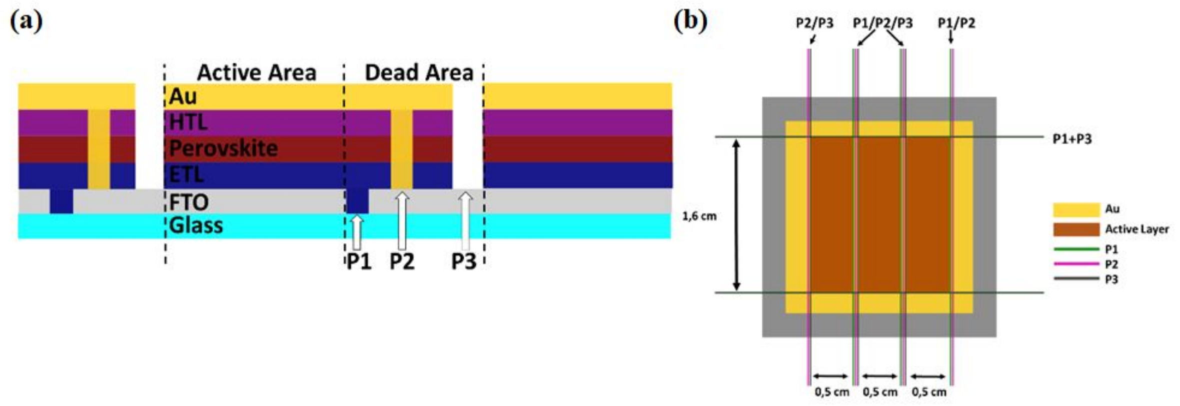

Figure S7. (a) Cross view of mini-module connections. (b) Front view of mini-module with a total active area of 2.4 cm^2.

Table S4. Summary of PEC systems for organic pollutant degradation.

Photoelectrode for PEC reactions	PEC reactions	Photocurrent (mA/cm^2)	References
Meso-$TiO_2/CsPbBr_3/Al_2O_3$	2-mercaptobenzothhiazole (MBT) oxidation	~3.0	current work
Al_2O_3/ITO:*$^{NCN}CN_x$	4-methylbenzyl alcohol oxidation	~1.4	(1)
TiO_2	Acid orange 7 oxidation	~0.06	(2)
Bi_2WO_6 nanoplate	Rhodamine B oxidation	~2.5	(3)
TiO_2 nanotubes	Atrazine oxidation	~0.38	(4)
BiO_x-TiO_2/Ti	Phenol oxidation	~0.25	(5)

*$^{NCN}CN_x$: cyanamide-functionalized carbon nitride

Table S5. A summarizing table of recent PEC reactions based on perovskite-modified electrodes.

PEC applications to a target chemical reaction	Contact electrode & structure	Perovskites used	Preparation method of perovskites	Protection layer	Cell Performance measured	Stability	References
Pollutant oxidation (MBT)	Meso-TiO_2 film (~1.6 μm)	Nano-$CsPbBr_3$ (separate sensitizer)	2-step Spin-coating (in-situ formation)	Al_2O_3	~3 mA/cm^2 (under 1sun)	~60% maintained after 30 min	current work

Pollutant oxidation (MBT)	Compact TiO_2 layer	Nano-$CsPbBr_3$ (making a connected film, ~60 nm thick)	Hot-injection (colloidal nanocrystal)	-	~0.15 mA/cm^2 (under 1sun)	~80% maintained after 2 min	(6)
Oxidation of benzyl alcohol (BzOH) to benzyl aldehyde (BzCHO)	Compact TiO_2 layer	Nano-$CsPbBr_3$ (making a connected film, ~60 nm thick))	Hot-injection (colloidal nanocrystal)	-	~0.04 mA/cm^2 (under 1sun)	~80% maintained after 2 min	(7)
Photodegradation of organic compound (curcumin)	Meso-TiO_2 film (~5.0 µm)	Nano-$CsPbBr_3$ (separate sensitizer)	Photocatalytic two-step method (in-situ formation)	-	~0.04 mA/cm^2 (under 1sun)	-	(8)
H_2O oxidation	Compact TiO_2 layer	Bulk-$CsPbBr_3$ (~50 nm thick)	2-step deposition process (spin-coating and dipping)	Meso-carbon layer (~20 µm thick) and graphite sheet (~25 µm thick)	~2.5 mA/cm^2 (under 1sun)	~58% maintained after 34 hours in an alkaline electrolyte	(9)
Synthesis of dimethoxydihydrofuran	Compact TiO_2 layer	$MAPbBr_3$ single-crystal film	Space-limited crystallization method	Al_2O_3/Ti	~7.8 mA/cm^2	~78% maintained after 6 hours	(10)

		(~14 µm thick)			(under 1sun)		
CO_2 reduction	FTO	Nano-$CsPbBr_3$ (making a connected film)	Hot-injection (colloidal nanocrystal)	Ni(tpy) catalyst	~7.0 mA/cm^2 (under 1sun)	~90% maintained after ~2 min.	(11)

REFERENCES

(1) Pulignani, C.; Mesa, C. A.; Hillman, S. A. J.; Uekert, T.; Giménez, S.; Durrant, J. R.; Reisner, E. Rational Design of Carbon Nitride Photoelectrodes with High Activity Toward Organic Oxidations. *Angew. Chem. Int. Ed.* **2022**, *61* (50), 202211587.

(2) Tantis, I.; Stathatos, E.; Mantzavinos, D.; Lianos, P. Photoelectrocatalytic Degradation of Potential Water Pollutants in the Presence of NaCl Using Nanocrystalline Titania Films. *J. Chem. Technol. Biotechnol.* **2015**, *90* (7), 1338–1344.

(3) Li, J.; Zhang, X.; Ai, Z.; Jia, F.; Zhang, L.; Lin, J. Efficient Visible Light Degradation of Rhodamine B by a Photo-Electrochemical Process Based on a Bi_2WO_6 Nanoplate Film Electrode. *J. Phys. Chem. C* **2007**, *111* (18), 6832–6836.

(4) Xie, S.; Tang, C.; Shi, H.; Zhao, G. Highly Efficient Photoelectrochemical Removal of Atrazine and the Mechanism Investigation: Bias Potential Effect and Reactive Species. *J. Hazard Mater.* **2021**, *415*.

(5) Park, H.; Bak, A.; Ahn, Y. Y.; Choi, J.; Hoffmannn, M. R. Photoelectrochemical Performance of Multi-Layered BiO_x-TiO_2/Ti Electrodes for Degradation of Phenol and Production of Molecular Hydrogen in Water. *J. Hazard Mater.* **2012**, *211–212*, 47–54.

(6) Cardenas-Morcoso, D.; Gualdrón-Reyes, A. F.; Ferreira Vitoreti, A. B.; García-Tecedor, M.; Yoon, S. J.; Solis De La Fuente, M.; Mora-Seró, I.; Gimenez, S. Photocatalytic and

Photoelectrochemical Degradation of Organic Compounds with All-Inorganic Metal Halide Perovskite Quantum Dots. *J. Phys. Chem. Lett.* **2019**, *10* (3), 630–636.

(7) Fernández-Climent, R.; Gualdrón-Reyes, A. F.; García-Tecedor, M.; Mesa, C. A.; Cárdenas-Morcoso, D.; Montanes, L.; Barea, E. M.; Mas-Marzá, E.; Julián-López, B.; Mora-Seró, I; Giménez, S. Switchable All Inorganic Halide Perovskite Nanocrystalline Photoelectrodes for Solar-Driven Organic Transformations. *Solar RRL* **2022**, *6*, 2100723.

(8) Gonzalez-Moya, J. R.; Chang, C. Y.; Radu, D. R.; Lai, C. Y. Photocatalytic Deposition of Nanostructured $CsPbBr_3$ Perovskite Quantum Dot Films on Mesoporous TiO_2 and Their Enhanced Visible-Light Photodegradation Properties. *ACS Omega* **2022**, *7* (30), 26738–26748.

(9) Poli, I.; Hintermair, U.; Regue, M.; Kumar, S.; Sackville, E. V.; Baker, J.; Watson, T. M.; Eslava, S.; Cameron, P. J. Graphite-Protected $CsPbBr_3$ Perovskite Photoanodes Functionalised with Water Oxidation Catalyst for Oxygen Evolution in Water. *Nat. Commun.* **2019**, *10* (1).

(10) Wang, X.-D.; Huang, Y.-H.; Liao, J.-F.; Wei, Z.-F.; Li, W.-G.; Xu, Y.-F.; Chen, H.-Y.; Kuang, D.-B. Surface Passivated Halide Perovskite Single-Crystal for Efficient Photoelectrochemical Synthesis of Dimethoxydihydrofuran. *Nat. Commun.* **2021**, *12* (1), 1202.

(11) Chen, Z.; Hu, Y.; Wang, J.; Shen, Q.; Zhang, Y.; Ding, C.; Bai, Y.; Jiang, G.; Li, Z.; Gaponik, N. Boosting Photocatalytic CO_2 Reduction on $CsPbBr_3$ Perovskite Nanocrystals by Immobilizing Metal Complexes. Chem. Mater. **2020**, *32* (4), 1517.
